# NEWS, SIRS and qSOFA criteria for predicting sepsis and sepsis with high risk of death in emergency room: Comparison and enhancement of sepsis prediction models in emergency care: Insights from CETAT and MIMIC-IV databases

**DOI:** 10.17305/bb.2024.11134

**Published:** 2024-11-05

**Authors:** Wenwen Wang, Kaipeng Wang, Yueguo Wang, Qingyuan Liu, Jian Sun, Ronghua Shi, Sicheng Liu, Huanli Wang, Yuan Yuan, Jun Xu, Kui Jin, Yixin Zhang

**Affiliations:** 1 Department of Emergency Medicine, The First Affiliated Hospital of USTC, Division of Life Sciences and Medicine, University of Science and Technology of China, Hefei, China; 2 School of Mathematics and Statistics, Nanjing University of Science and Technology, Nanjing, China; 3 School of Mathematics and Physics, Anhui Jianzhu University, Hefei, China; 4 HKU Business School, The University of Hong Kong, Hong Kong, China; 5 School of Mathematical Sciences, University of Science and Technology of China, Hefei, China; 6 Information Technology Department, The First Affiliated Hospital of USTC, Division of Life Sciences and Medicine, University of Science and Technology of China, Hefei, China; 7 Department of Emergency Medicine, Peking Union Medical College Hospital, Chinese Academy of Medical Sciences & Peking Union Medical College, Beijing, China

**Keywords:** Sepsis, emergency department, Chinese Emergency Triage Evaluation and Treatment (CETAT), Medical Information Mart for Intensive Care (MIMIC)-IV, scoring system

## Abstract

Early identification of sepsis in emergency department patients is critical for initiating timely interventions, highlighting the need for effective predictive scoring systems. A retrospective observational study was conducted using data from the CETAT database collected between December 2019 and October 2021. The study evaluated how well the systemic inflammatory response syndrome (SIRS), quick Sepsis-related Organ Failure Assessment (qSOFA), and National Early Warning Score (NEWS) scoring systems, along with logistic regression models, predict sepsis, and high-risk sepsis in emergency department patients. The logistic regression models were further optimized by incorporating additional features based on local data. A total of 12,799 patients were analyzed, including 1360 sepsis cases, of which 373 were classified as high-risk sepsis. The NEWS score demonstrated superior predictive performance compared to qSOFA and SIRS, with an area under the receiver operating characteristic curve (AUC-ROC) of 0.737 (95% confidence interval [CI] 0.72–0.75) for sepsis and 0.653 (95% CI 0.62–0.69) forhigh risk sepsis. After optimization, the NEWS-based model improved to an AUC-ROC of 0.756 (95% CI 0.74–0.77) for sepsis and 0.718 (95% CI 0.69–0.75) for high-risk sepsis. Further enhancement was observed with the inclusion of additional clinical variables, resulting in AUC-ROC values of 0.834 (95% CI 0.82–0.85) for sepsis and 0.756 (95% CI 0.73–0.78) for high-risk sepsis. Data from the Medical Information Mart for Intensive Care (MIMIC)-IV database, which included sepsis status and relevant variables for SIRS, qSOFA, and NEWS score calculations, confirmed that the optimized NEWS-based model improved the sepsis prediction AUC-ROC from 0.690 (95% CI 0.68–0.70) to 0.708 (95% CI 0.70–0.72), and consistently outperformed qSOFA and SIRS in sepsis prediction.

## Introduction

Approximately 20 million patients worldwide are diagnosed with sepsis annually, resulting in over 5 million deaths [1–3]. The emergency department serves as the initial point of access for patients presenting with sepsis. Patients with septic conditions in emergency settings frequently exhibit rapid disease progression, and clinicians often have limited clinical data at their disposal [4–6]. Accurate assessment of disease severity, optimized management, and early diagnosis of sepsis are significantly associated with patient prognosis [7]. However, achieving these objectives in clinical practice is fraught with challenges. Developing an appropriate model for diagnosing sepsis and assessing its severity in the emergency department holds considerable clinical and social importance. Currently, there are no globally standardized diagnostic approaches. In 1991, the American College of Chest Physicians (ACCP) and the American Society of Critical Care Medicine (SCCM) introduced the systemic inflammatory response syndrome (SIRS) criteria for the diagnosis of sepsis [8, 9]. Nonetheless, subsequent studies have indicated that the sensitivity and specificity of these criteria are insufficient. In 2016, the quick Sepsis-related Organ Failure Assessment (qSOFA) was proposed as a replacement for SIRS in the early diagnosis of sepsis [10]. However, it has been noted that the accuracy of qSOFA remains inadequate in clinical practice [11–14]. In the revised Sepsis Guidelines of 2021, qSOFA is no longer recommended as a standalone diagnostic tool for sepsis [15, 16]. The National Early Warning Score (NEWS) was introduced in 2012 for early risk evaluation among emergency patients, although it was not originally designed for patients with sepsis. Recent studies have demonstrated that NEWS can also be employed to predict disease severity among emergency patients with sepsis [17–20].

A critical consideration in understanding the disparate research findings regarding these scoring systems is the significant heterogeneity among septic patients, including demographic characteristics, variations in healthcare systems, and divergent treatment protocols across different countries [7]. Consequently, achieving satisfactory predictive and evaluative accuracy using a uniform scoring system for heterogeneous septic patients presents significant challenges [11, 13, 21]. To address the specific needs of local clinical practice, it may be necessary to update and optimize the scoring system based on local patient data.

In this study, we analyzed the Chinese Emergency Triage Evaluation and Treatment (CETAT) database, developed by the Chinese Emergency Medicine Partnerships (CEMP), to evaluate the diagnosis and severity assessment of sepsis in emergency patients. The aim of this study was to assess the potential benefits of updating and optimizing the qSOFA, SIRS, and NEWS scoring systems using local patient data. To facilitate a meaningful comparison, relevant data were also extracted from the Medical Information Mart for Intensive Care (MIMIC)-IV database and analyzed in a similar manner for sepsis diagnosis. By utilizing the additional clinical features available in the CETAT database, we also constructed predictive models to explore the possibility of enhancing predictive power further.

## Materials and methods

### Study design

A retrospective observational study was conducted in the emergency departments of two medical centers within a tertiary academic hospital in China. This study utilized data from the CETAT, covering the period from December 2019 to October 2021, and received approval from the ethics committee of the First Affiliated Hospital of the University of Science and Technology of China (USTC), approval number 2021-ky027. Data were also retrieved from the MIMIC-IV database. The hospital ethics committee waived the requirement for informed consent due to the study’s observational nature. No commercial support was provided for this project.

### Data

This study was based on data from the CETAT database, which was created and recently updated by the CEMP. A detailed description of the CETAT database is available in our previous study [11]. Our study included patients admitted to the emergency departments of two clinical centers: the First Affiliated Hospital of Anhui Provincial Hospital and the Southern District of Anhui Provincial Hospital (Anhui Cardiovascular and Cerebrovascular Hospital). The School of Mathematical Sciences at USTC is responsible for data coordination and maintenance. Personal privacy information was removed prior to data usage. For comparative analysis, relevant data from the MIMIC-IV database were also retrieved, focusing on patients admitted to the intensive care unit (ICU) from the emergency department, with or without a confirmed sepsis diagnosis.

### Inclusion/exclusion criteria

Based on the updated CETAT database, the inclusion criteria were defined as follows: patients aged 18 years or older; patients who did not require palliative or restrictive treatment following admission; patients who did not experience cardiac arrest outside of the hospital; and patients with complete data available for the calculation of SIRS, qSOFA, and NEWS scores. Conversely, the exclusion criteria included patients younger than 18 years, patients who departed the emergency department prior to receiving complete treatment, and patients with incomplete data necessary for the calculation of SIRS, qSOFA, and NEWS scores.

### Definitions and outcomes

Sepsis was diagnosed by the treating physicians upon the patient’s admission to the emergency department. The primary outcome of the study was the diagnosis of sepsis at the time of admission to the emergency department. The secondary outcome was sepsis with a high risk of mortality (Risk^+^), defined as patients requiring admission to an ICU, which includes general ICU, surgical ICU, cardiac ICU, and emergency ICU, or patients who died in the emergency department. Utilizing the clinical information obtained from the CETAT database, the SIRS, qSOFA, and NEWS scores were calculated for each patient (Table 1), adhering to the corresponding scoring criteria.

**Table 1:** Characteristics of study population based on CETAT database, stratified by sepsis status

**Characteristic**	**Sepsis^−^**	**Sepsis^+^**	***P* value**	**OR**	OR*
	***N* ═ 11,439**	***N* ═ 1360**			
Male	7216 (63.1%)	863 (63.5%)	0.81	0.98 (0.88, 1.11)	–
Age, median (IQR)	63.0 (51.0, 73.0)	70.0 (54.0, 80.0)	<0.01	1.02 (1.01, 1.02)	–
Transported by ambulance	5473 (47.8%)	866 (63.7%)	<0.01	0.52 (0.47, 0.59)	0.53 (0.47, 0.59)
Coma	1829 (16.0%)	256 (18.8%)	<0.01	1.22 (1.05, 1.40)	1.23 (1.06, 1.42)
Body temperature, median (IQR)	36.5 (36.3, 36.6)	36.5 (36.3, 37.0)	<0.01	1.83 (1.70, 1.97)	1.89 (1.76, 2.04)
Heart rate, median (IQR)	81.0 (72.0, 96.0)	97.5 (82.0, 115.0)	<0.01	1.02 (1.02, 1.03)	1.03 (1.02, 1.03)
Respiration rate, median (IQR)	20.0 (20.0, 20.0)	21.0 (20.0, 24.0)	<0.01	1.15 (1.13, 1.17)	1.14 (1.12, 1.16)
Systolic pressure, median (IQR)	141.0 (121.0, 163.0)	129.0 (111.0, 151.0)	<0.01	0.99 (0.99, 0.99)	0.99 (0.99, 0.99)
Diastolic pressure, median (IQR)	83.0 (71.0, 96.0)	77.0 (65.0, 89.0)	<0.01	0.98 (0.98, 0.99)	0.98 (0.98, 0.99)
SpO2, median (IQR)	97.0 (95.0, 98.0)	95.0 (88.0, 97.0)	<0.01	0.97 (0.97, 0.98)	0.97 (0.97, 0.98)
Death	113 (1.0%)	44 (3.2%)	<0.01	3.35 (2.33, 4.73)	2.89 (2.00, 4.09)
WBC, median (IQR)	9.3 (6.9, 12.7)	10.6 (7.2, 14.6)	<0.01	1.01 (1.01, 1.01)	1.01 (1.01, 1.02)
NE%, median (IQR)	80.2 (70.2, 87.3)	85.0 (76.9, 90.2)	<0.01	1.03 (1.03, 1.04)	1.03 (1.03, 1.04)
HGB, median (IQR)	129.0 (111.0, 143.0)	120.0 (99.0, 138.0)	<0.01	0.99 (0.99, 0.99)	0.99 (0.99, 0.99)
PLT, median (IQR)	180.0 (139.0, 227.0)	183.0 (133.0, 246.0)	0.19	1.0009 (1.0003, 1.0015)	1.0013 (1.0007, 1.0019)
ALT, median (IQR)	20.6 (14.0, 33.3)	24.0 (14.0, 49.1)	<0.01	1.0003 (1.0001, 1.0005)	1.0003 (1.0002, 1.0005)
AST, median (IQR)	25.0 (19.0, 38.0)	30.0 (20.0, 56.9)	<0.01	1.0001 (1.0001, 1.0002)	1.0001 (1.0001, 1.0002)
Albumin, median (IQR)	40.8 (36.9, 44.0)	35.9 (31.3, 40.2)	<0.01	0.90 (0.89, 0.91)	0.90 (0.90, 0.91)
TBIL, median (IQR)	12.3 (8.6, 18.1)	13.6 (8.8, 23.1)	<0.01	1.01 (1.01, 1.01)	1.01 (1.01, 1.01)
Creatinine, median (IQR)	70.0 (55.0, 91.0)	78.0 (57.8, 121.0)	<0.01	1.0009 (1.0006, 1.0012)	1.0009 (1.0006, 1.0012)
CO2CP, median (IQR)	23.5 (21.0, 25.9)	22.4 (18.9, 25.9)	<0.01	0.97 (0.96, 0.98)	0.97 (0.96, 0.98)
Potassium, median (IQR)	3.9 (3.6, 4.2)	4.0 (3.6, 4.5)	<0.01	1.28 (1.19, 1.38)	1.24 (1.15, 1.34)
Sodium, median (IQR)	139.0 (136.9, 141.0)	138.0 (134.1, 141.0)	<0.01	0.95 (0.94, 0.96)	0.95 (0.94, 0.97)
Phosphorus, median (IQR)	1.1 (0.9, 1.3)	1.1 (0.9, 1.4)	<0.01	1.35 (1.22, 1.50)	1.37 (1.23, 1.52)
AG, median (IQR)	12.8 (9.6, 16.5)	16.2 (13.2, 19.6)	<0.01	1.09 (1.08, 1.10)	1.09 (1.08, 1.10)
Osmotic pressure, median (IQR)	282.6 (277.8, 288.1)	283.1 (275.3, 291.5)	0.76	1.0028 (0.9996 1.0061)	1.0014 (0.9979, 1.0046)
Glucose, median (IQR)	7.3 (6.1, 9.4)	7.3 (6.0, 9.7)	0.88	1.0108 (0.9999, 1.0213)	1.0084 (0.9971, 1.0191)
Prothrombin time, median (IQR)	13.2 (12.5, 14.1)	13.5 (12.4, 14.9)	<0.01	1.012 (1.004, 1.019)	1.011 (1.003, 1.018)
APTT, median (IQR)	34.0 (30.2, 37.8)	34.6 (28.9, 39.9)	0.02	1.008 (1.002, 1.012)	1.007 (1.002, 1.011)
Fibrinogen, median (IQR)	3.2 (2.5, 4.1)	4.1 (2.9, 5.5)	<0.01	1.38 (1.34, 1.43)	1.38 (1.34, 1.43)
TT, median (IQR)	17.2 (16.3, 18.2)	16.8 (15.7, 17.9)	<0.01	1.0006 (0.9952, 1.0049)	0.9994 (0.9939, 1.0038)
High risk of death	1993 (17.4%)	373 (27.4%)	<0.01	1.79 (1.57, 2.04)	1.79 (1.57, 2.04)
qSOFA, median (IQR)	0.0 (0.0, 1.0)	1.0 (0.0, 1.0)	<0.01	2.32 (2.14, 2.52)	2.32 (2.14,2.52)
SIRS, median (IQR)	1.0 (0.0, 1.0)	2.0 (1.0, 2.0)	<0.01	2.15 (2.04, 2.27)	2.23 (2.10, 2.35)
NEWS, median (IQR)	2.0 (0.0, 4.0)	5.0 (3.0, 7.0)	<0.01	1.25 (1.23, 1.27)	1.25 (1.23, 1.27)

CETAT: Chinese emergency triage evaluation and treatment; IQR: Interquartile range; NEWS: National early warning score; qSOFA: Sepsis-related organ failure assessment; PLT: Platelet; TBIL: Total bilirubin; SIRS: Systemic inflammatory response syndrome; AG: Anion gap.

### Ethical statement

This study was approved by the Ethics Committee of the First Affiliated Hospital of USTC, Hefei, China (Approval Number: 2021-ky027).

### Statistical analysis

Descriptive statistics were used to summarize the continuous variables, with the median and interquartile range (IQR) reported. The Mann–Whitney–Wilcoxon rank sum test was utilized for analysis. Categorical variables were expressed as frequencies and percentages, and comparisons were made using Fisher’s exact test. Odds ratios (ORs) were calculated through logistic regression to assess associations between characteristics and the risk of sepsis or high risk of death. Adjusted ORs were also calculated, with age and sex included as confounders in the CETAT dataset, and age included as a confounder in the MIMIC-IV dataset. The predictive performance of the scoring systems and logistic regression models was evaluated using the area under the receiver operating characteristic curve (AUC-ROC). Logistic regression was also used to optimize the scoring systems and construct additional predictive models. To achieve parsimonious models, a stepwise variable selection method was applied based on either Akaike’s information criterion (AIC) or a predetermined *P* value threshold. Finally, variance inflation factor (VIF) tests were conducted to identify and mitigate multicollinearity issues.

## Results

### Enrollment and baseline characteristics

In total, 12,799 patients were included in this study (Figure S1). Among these, 1360 patients were diagnosed with sepsis (sepsis^+^, as presented in Table 1), and 373 (27.4%) were identified as patients with sepsis at high risk of death (risk^+^, as shown in Table S1). Compared to patients without sepsis (sepsis^−^), those with sepsis were older (median age: 70 years; interquartile range IQR: 54–80), more likely to be comatose and transported by ambulance, and exhibited elevated heart rates, respiratory rates, and body temperatures, in addition to lower blood pressure and blood oxygen saturation (all *P* values < 0.01, as indicated in Table 1). Patients with sepsis also presented higher white blood cell counts, neutrophil counts, liver enzyme levels, creatinine levels, and anion gap (AG), alongside lower levels of albumin and sodium ions (all *P* < 0.01, as detailed in Table 1). Furthermore, the SIRS, qSOFA, and NEWS scores were significantly higher in patients with sepsis (all *P* < 0.01, as reported in Table 1). For comparative purposes, data from 35,017 patients (32,872 sepsis-and 2145 sepsis+ patients) were retrieved from the MIMIC-IV database, demonstrating similar baseline characteristics to our study data (Table S2).

In comparison to septic patients without a high risk of death, those classified as septic risk^+^ exhibited a higher proportion of coma, elevated heart rates, lower systolic and diastolic blood pressure, increased white blood cell counts, higher liver enzyme levels, total bilirubin (TBIL), and AG, as well as lower albumin and platelet (PLT) levels (all *P* values < 0.05, as detailed in Table S1). Additionally, their SIRS, qSOFA, and NEWS scores were also significantly higher (all *P* < 0.01, as indicated in Table S1).

As presented in Table 1, the ORs and 95% confidence intervals (CIs) for coma, body temperature, respiratory rate, mortality, potassium levels, and high risk of death exhibited a positive association with sepsis. Conversely, a negative association between transportation by ambulance and sepsis was observed, with an OR of 0.52 (95% CI: 0.47, 0.59). The SIRS, qSOFA, and NEWS scores all demonstrated a positive association with sepsis, yielding ORs of 2.23, 1.19, and 1.02, respectively. After adjustment for potential confounders, such as age and sex, the ORs continued to indicate consistent associations between these characteristics and sepsis. Patients from the CETAT dataset exhibited a similar association between characteristics and sepsis, particularly in relation to a high risk of death (Table S1). Additionally, the adjusted OR was calculated using age as a confounding variable in the MIMIC-IV dataset. Overall, the scores demonstrated a similar association between baseline characteristics and sepsis in comparison to our data (Table S2).

### Prediction of diagnosis and severity of sepsis with SIRS, qSOFA, and NEWS scores

**Figure 1.**
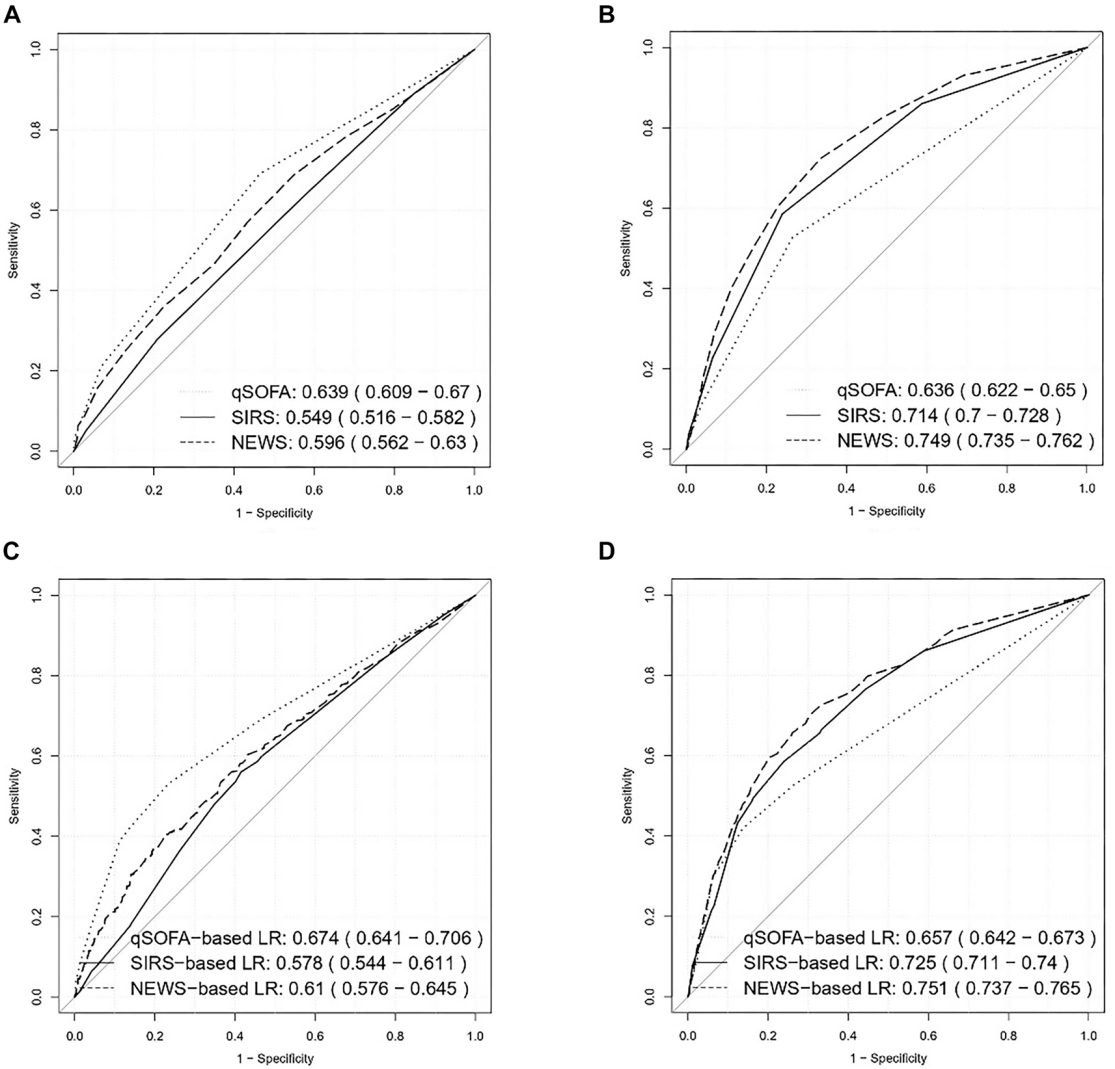
Prediction performance (ROC) for the prediction of sepsis (among study population, [A and C]) and the prediction of sepsis with high risk of death (among patients with sepsis, [B and D]), based on medical scoring system (A and B) or logistic regression with the available features in medical scoring system (C and D). (Data from CETAT database.) For each evaluation, the AUC-ROC with 95% confidence interval are provided. NEWS: National early warning score; qSOFA: Sepsis-related organ failure assessment; SIRS: Systemic inflammatory response syndrome; CETAT: Chinese emergency triage evaluation and treatment; ROC: Receiver operating characteristic; AUC-ROC: Area under the receiver operating characteristic curve.

For patients with sepsis in the CETAT database, the median scores for qSOFA, SIRS, and NEWS were 1.0 (IQR [0.0, 1.0]), 2.0 (IQR [1.0, 2.0]), and 5.0 (IQR [3.0, 7.0]), respectively (Table 1). In terms of sepsis prediction, the AUC-ROC was 0.71 (95% CI [0.70, 0.73]) for SIRS, 0.64 (95% CI [0.62, 0.65]) for qSOFA, and 0.74 (95% CI [0.72, 0.75]) for NEWS. Both SIRS and NEWS exhibited significantly larger AUC-ROCs than qSOFA (*P* < 0.001) (Figure 1A, Table 3). In comparison, for sepsis prediction based on MIMIC-IV data, the AUC-ROC was 0.69 (95% CI [0.68, 0.70]) for SIRS, 0.62 (95% CI [0.61, 0.63]) for qSOFA, and 0.69 (95% CI [0.68, 0.70]) for NEWS (Figure S2A and Table S3).

**Table 2:** The prediction performance of sepsis and sepsis with high risk of death with scoring systems

**Prediction of sepsis**	**N**	**AUC-ROC**	**95% CI**	***P* value**	
qSOFA	12,799	0.636	0.622	0.650	Reference
SIRS	12,799	0.714	0.700	0.728	<0.001
NEWS	12,799	0.737	0.723	0.750	<0.001
**Prediction of sepsis with high risk of death**	**N**	**AUC-ROC**	**95% CI**	***P* value**	
qSOFA	1360	0.639	0.609	0.670	Reference
SIRS	1360	0.549	0.516	0.582	<0.001
NEWS	1360	0.653	0.620	0.686	0.262

Prediction performance (AUC-ROC, with 95% confidence interval) for the prediction of sepsis (among study population, first scenario) and the prediction of sepsis with high risk of death (among patients with sepsis, second scenario), based on medical scoring system. (Data from CETAT database.) For each prediction scenario, the AUC-ROC for qSOFA was considered as the reference for comparison (to obtain test *P* value). NEWS: National early warning score; qSOFA: Sepsis-related organ failure assessment; CETAT: Chinese emergency triage evaluation and treatment; AUC-ROC: Area under the receiver operating characteristic curve; SIRS: Systemic inflammatory response syndrome.

**Table 3:** Sensitivity and specificity of scoring systems updated with additional clinical features

**Prediction of sepsis**	**AUC-ROC**	**95% CI**		***P* value**
Baseline nine-variable model	0.7682	0.7546	0.7817	Reference
Stepwise-selected (AIC) model	0.8344	0.8236	0.8452	<0.001
Stepwise-selected (*P* < 0.1) model	0.8039	0.7914	0.8164	<0.001
Stepwise-selected (*P* < 0.05) model	0.8023	0.7898	0.8149	<0.001
Stepwise-selected (*P* < 0.01) model	0.796	0.783	0.8089	<0.001
*Prediction of sepsis with high risk of death*				
Baseline nine-variable model	0.7224	0.6913	0.7534	Reference
Stepwise-selected (AIC) model	0.7526	0.7234	0.7818	<0.001

Prediction performance (AUC-ROC, with 95% confidence interval) for the prediction of sepsis (among study population, first scenario) and the prediction of sepsis with high risk of death (among patients with sepsis, second scenario), based on logistic regression with additional clinical features. (Data from CETAT database.) For each prediction scenario, the AUC-ROC for baseline nine-variable model was considered as the reference for comparison (to obtain test *P* value). CETAT: Chinese emergency triage evaluation and treatment; AUC-ROC: Area under the receiver operating characteristic curve; CI: Confidence interval; AIC: Akaike information criterion.

For the scoring systems (SIRS, qSOFA, or NEWS) evaluated with logistic regression and specified features, the systems were optimized using local patient data. The AUC-ROCs were significantly improved: 0.73 (95% CI [0.71, 0.74]) for SIRS, 0.66 (95% CI [0.64, 0.67]) for qSOFA, and 0.76 (95% CI [0.74, 0.77]) for NEWS (Figure 1C, Table S4). Similarly, the performance of the optimized SIRS, qSOFA, and NEWS for predicting sepsis with a high risk of mortality was significantly enhanced compared to their original scoring systems: 0.58 (95% CI [0.54, 0.61]) vs 0.55 (95% CI [0.52, 0.58]) for SIRS, 0.67 (95% CI [0.64, 0.71]) vs 0.64 (95% CI [0.61, 0.67]) for qSOFA, and 0.72 (95% CI [0.69, 0.75]) vs 0.65 (95% CI [0.62, 0.69]) for NEWS (Figure 1B and 1D, Table 2, and Table S4). This improvement was further validated by the Delong test (*P* < 0.001).

For sepsis prediction, the scoring systems (SIRS, qSOFA, and NEWS) were also optimized using local patient data from the MIMIC-IV database. This optimization led to significantly improved AUC-ROCs: 0.69 (95% CI [0.68, 0.71]) for SIRS, 0.63 (95% CI [0.61, 0.64]) for qSOFA, and 0.71 (95% CI [0.70, 0.72]) for NEWS (Figure S2B and Table S3).

### Construct logistic regression model by stepwise method

With the incorporation of additional patient clinical features from the recent CETAT database, further logistic regression models could be developed to investigate whether predictive accuracy could be enhanced. Nine baseline measurements/variables, including gender, blood pressure, and body temperature, were readily available at admission and can be easily collected in a general practice setting without the need for specialized equipment. In contrast, forty-three additional measurements/variables were accessible after admission; however, the collection of these data typically necessitates blood samples and associated equipment.

Initially, a logistic regression model was constructed utilizing the nine baseline variables. Subsequently, additional variables were incorporated into the model through a stepwise variable selection procedure employing various *P* value thresholds (0.01, 0.05, or 0.1). Alternatively, a stepwise variable selection procedure was conducted using the Akaike Information Criterion (AIC).

**Figure 2.**
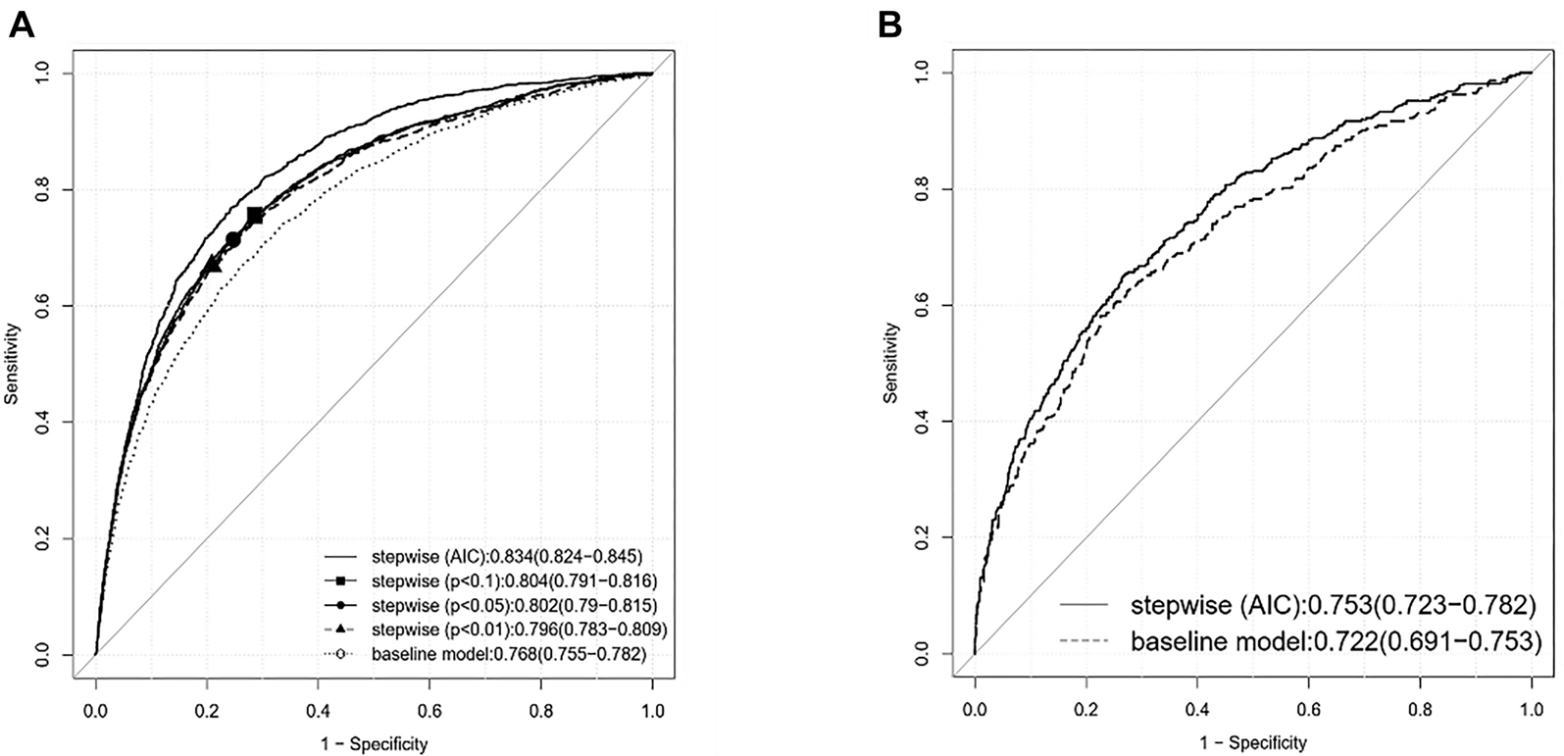
Prediction performance (ROC) for the prediction of sepsis (among study population, [A]) and the prediction of sepsis with a high risk of death (among patients with sepsis, [B]), based on logistic regression with additional clinical features. (Data from CETAT database.) For each evaluation, the AUC-ROC with 95% confidence intervals are provided. CETAT: Chinese emergency triage evaluation and treatment; AIC: Akaike information criterion; ROC: Receiver operating characteristic; AUC-ROC: Area under the receiver operating characteristic curve.

For sepsis prediction, AUC-ROC of the nine-variable baseline model was found to be 0.77 (95% CI [0.76, 0.78]), which demonstrated statistical superiority over the AUC-ROC for the NEWS, as well as for SIRS and quick Sequential Organ Failure Assessment (qSOFA) scores (*P* < 0.001), according to the Delong test. Utilizing AIC for stepwise variable selection resulted in a thirty-four-variable model. Following the VIF test to evaluate multicollinearity within this model, a refined thirty-one-variable model was identified, achieving an AUC-ROC as high as 0.83 (95% CI [0.82, 0.85]). When various *P* value thresholds were applied in the stepwise variable selection, the corresponding performance (i.e., AUC-ROC) fell between those of the two models (Figure 2A, Table 3). Specifically, when employing *P* value thresholds of 0.1, 0.05, and 0.01, the model comprised twenty nine, twenty six, and nineteen variables, respectively. The maximum VIF values for the three models selected based on *P* value were all below 10, indicating the absence of multicollinearity among these models. The variables selected through different criteria are detailed in Table S9.

Subsequently, for predicting sepsis with a high risk of mortality, the AUC-ROC for the baseline nine-variable model was 0.72 (95% CI [0.69, 0.75]), which was statistically superior to the AUC-ROC for NEWS, SIRS, and qSOFA (*P* < 0.001) based on the Delong test. When AIC was employed in the stepwise variable selection, a fifteen-variable model was established, achieving an AUC-ROC of 0.75 (95% CI [0.72, 0.78]) after addressing issues of multicollinearity. In this analysis, no discrepancies were observed based on varying *P* value thresholds for stepwise variable selection (Figure 2B, Table S5). The six additional variables selected are presented in Table S9.

### Sensitivity and specificity

When the default cutoff value was applied to each scoring system, the corresponding sensitivity and specificity values were computed (Table S5 and S6). The sensitivity of both the SIRS and the quick Sequential Organ Failure Assessment (qSOFA) was notably low, although satisfactory specificity was achieved. In contrast, the NEWS exhibited a relatively higher sensitivity, but its specificity was lower than that of SIRS and qSOFA.

For a locally optimized scoring system, specifically a predictive logistic regression model, the default cutoff value becomes inapplicable because the output is expressed as a probability value ranging from 0 to 1. Given that the associated Receiver Operating Characteristic (ROC) curves were relatively low (Figure 1C and 1D), we determined the cutoff value for this probability by maximizing the sum of sensitivity and specificity (Tables S7 and S8). Overall, it proved challenging for both sensitivity and specificity to attain satisfactory performance levels (i.e., >80%). We also assessed the sensitivity and specificity of the reported predictive logistic regression models incorporating additional patient clinical features. The associated ROC curves indicated relatively high performance (Figure 2); thus, we selected the cutoff value for the predicted probability based on the following relevant clinical scenarios. In the context of sepsis prediction, a specificity of no less than 80% was associated with a relatively high true negative rate, resulting in sensitivity values of approximately 60%–70% (Table 4). Conversely, for predicting sepsis with a high risk, a sensitivity threshold of no less than 80% was attained, which corresponded to specificity values ranging from approximately 45%–55% (Table 4).

**Table 4:** Sensitivity and specificity of scoring systems updated with additional clinical features

**Prediction of sepsis**	**Sensitivity**	**Specificity**
Baseline nine-variable model	58.9%	80.0%
Stepwise-selected (AIC) model	71.7%	80.0%
Stepwise-selected (*P* < 0.1) model	66.6%	80.0%
Stepwise-selected (*P* < 0.05) model	66.2%	80.0%
Stepwise-selected (*P* < 0.01) model	65.3%	80.0%
**Prediction of sepsis with high risk of death**	**Sensitivity**	**Specificity**
Baseline nine-variable model	80.2%	44.8%
Stepwise-selected (AIC) model	80.2%	55.02%

Sensitivity and specificity for the prediction of sepsis (among study population, first scenario) and the prediction of sepsis with high risk of death (among patients with sepsis, second scenario), based on logistic regression with additional clinical features. (Data from CETAT database.) CETAT: Chinese emergency triage evaluation and treatment; AIC: Akaike information criterion.

## Discussion

### Main contributions

Our analysis revealed that the NEWS exhibited superior predictive value compared to the SIRS and Quick Sequential Organ Failure Assessment (qSOFA) scores in predicting both sepsis and sepsis with a high risk of mortality. These findings suggest that the parameters included in the current scoring system are both reasonable and effective. Furthermore, our results indicate that the accuracy of predictions could be further enhanced by incorporating local data and additional clinically relevant features. This study was conducted on a large emergency sample from the Asia–Pacific region, providing valuable evidence-based medical insights for the accurate triage and evaluation of emergency patients with sepsis within this specific geographical context. These findings underscore the importance of utilizing the NEWS score as a valuable tool in the assessment and management of patients with sepsis, thereby contributing to improved patient outcomes and informing clinical decision-making in emergency departments within the Asia–Pacific region.

### Relationship to other studies

The diagnosis and severity assessment of sepsis remain areas needing improvement, with findings from various studies often lacking consistency. Initially, SIRS was used as a diagnostic criterion for sepsis. However, early studies indicated that the specificity and sensitivity of the SIRS criteria were insufficient, limiting its applicability in clinical practice [22]. Usman et al. [18] demonstrated that NEWS, SIRS, and qSOFA have high predictive values for sepsis, with NEWS showing the highest accuracy (AUC-ROC values of 0.91, 0.88, and 0.81, respectively). Similarly, Oduncu et al. [23] found that AUC-ROC values for predicting sepsis were 0.73 for NEWS, 0.57 for SIRS, and 0.73 for qSOFA. Regarding the prediction of disease severity, Gole et al. [14] found that qSOFA was not superior to SIRS in predicting ICU mortality among patients with an initial diagnosis of sepsis; the AUC-ROC for qSOFA was approximately 0.60. Additionally, Goulden et al. [17] found that NEWS and qSOFA had similar predictive values (AUC-ROC: 0.62) for in-hospital death among patients with sepsis, while SIRS had a significantly lower AUC-ROC (0.49). These varied findings highlight ongoing debate and inconsistencies in sepsis assessment and prediction using different scoring systems, underscoring the need for further research and standardization to establish more accurate and reliable tools for sepsis diagnosis and severity assessment.

Several factors contribute to the variability observed across studies in predicting sepsis diagnosis and prognosis. First, differences in diagnostic criteria for sepsis play a significant role. For instance, Omar et al. defined sepsis as an increase in SIRS criteria accompanied by elevated lactate levels, decreased blood pressure, and lack of improvement after appropriate fluid resuscitation. In contrast, other studies have defined sepsis based on physician judgment, leading to variations in predictive efficacy. Second, differences in population characteristics across countries can impact results. The predictive value of qSOFA has been reported to differ between developed and developing countries [13, 24]. For example, in approximately one-third of cases, patients were not admitted to the emergency department due to limited medical resources, potentially introducing bias into the study. Third, patient severity in different studies may vary due to the lack of effective triage systems in many developing countries. Compared to Usman et al. [18], the scores in our study were generally lower. This difference in patient severity may contribute to relatively less accurate predictions of high-risk patients; similar findings were observed in a study in Thailand [25].

Finally, this study utilized patient data from the MIMIC-IV database, which primarily includes patients admitted to the EICU. There may be potential data heterogeneity between this database and other data sources, such as differences in how data were collected, processed, or recorded. Our data were specifically derived from emergency department cases, which may also contribute to discrepancies. Additionally, predictive accuracy can be significantly improved when optimized for local data and by incorporating clinical features available later in the patient’s treatment course. Consequently, different populations may require localized or newly developed scoring systems to enhance the accuracy of sepsis prediction and prognosis assessment.

### Limitations

This study has several limitations. First, the data used in this study were not specifically collected for the analysis of patients with sepsis. The qSOFA, SIRS, and NEWS criteria applied in the emergency department were based on the patients’ initial examination results at admission, and the timing of sepsis diagnosis varied across patients. As a result, we could not track changes in these scores over the course of illness. Second, although the study included data from over 10,000 emergency department patients, nearly half of the cases had to be excluded due to missing values. This exclusion may have introduced potential bias and limited the generalizability of our findings. Third, the new model included parameters that require specific point-of-care testing (POCT) methods, such as AG and PLT distribution width. However, these parameters may not be readily available in community health settings, primary hospitals, or rural areas in developing countries. Fourth, the data used in this study were collected in an emergency department setting, which made it difficult to track patient outcomes after transfer to the ICU or general wards. The lack of long-term follow-up data limits our ability to assess the models’ performance beyond the initial emergency department context. Additionally, models constructed in the emergency department often prioritize sensitivity, whereas models developed during hospitalization may be optimized for specificity. This distinction should be carefully considered when applying these models outside the emergency department. Lastly, our models were developed using the entire CETAT dataset, which presents a risk of overfitting due to the lack of cross-validation or an external validation dataset. While logistic regression models can still be clinically useful when rigorous statistical criteria and clinical reasoning are applied, even with imbalanced datasets and without cross-validation, future research should aim to collect additional external datasets to validate the models, confirm their generalizability, and reduce the risk of overfitting.

## Conclusion

NEWS is preferred over SIRS and qSOFA for predicting sepsis, particularly in cases with a high risk of mortality in emergency department settings. Local data suggests that updating or optimizing current medical scoring systems—or even creating new ones—might be necessary to enhance predictive accuracy further.

## Supplemental data

Supplementary data are available at the following link: https://www.bjbms.org/ojs/index.php/bjbms/article/view/11134/3566.

## Data Availability

Data are available on request to the corresponding authors.
